# Revisiting minor millets as an underutilised super food for enduring global food security

**DOI:** 10.1016/j.fochx.2026.103613

**Published:** 2026-02-02

**Authors:** Moumita Das, Arpita Das, Harsh B. Jadhav, Dheeraj Kumar, Aayeena Altaf, Pankaj B. Pathare, Robert Mugabi

**Affiliations:** a Department of Food and Nutrition, Swami Vivekananda University, Barrackpore, West Bengal, India; b Brainware University, Department of Food and Nutrition, Barasat, West Bengal, India; c Food Technology, Amity Institute of Biotechnology, Amity University, Rajasthan, India; d Department of Soils, Water and Agricultural Engineering, College of Agricultural & Marine Sciences, Sultan Qaboos University, Muscat 123, Oman; e Department of Food Technology and Nutrition, Makerere University, Kampala, Uganda

**Keywords:** Minor millets, Underutilised crops, Food security, Nutrition security

## Abstract

The growing global dependence on major cereals such as rice and wheat has intensified concerns over food and nutrition security, particularly among marginalized populations facing supply and climate challenges. This underscores the urgent need to diversify cropping systems by promoting resilient alternatives. Minor millets including finger, foxtail, proso, barnyard, kodo, and little millets represent a group of underutilised cereals with exceptional adaptability to harsh environmental conditions and low-input agriculture. This review synthesizes recent findings on the agronomic, nutritional, and socio-economic potential of minor millets as sustainable staple crops in food-insecure and climate-vulnerable regions. Despite being a synthesis of existing studies, this review offers a *novel integrative perspective* by re-evaluating previous research through the lens of climate resilience, nutritional security, and livelihood improvement. It emphasizes underexplored aspects such as the comparative adaptability of different millet species and their role in localized food systems providing actionable insights for research, and development.

## Highlights

• Minor millets can be considered as ‘super foods’ or ‘smart foods’ because they are a powerhouse of nutrition.
• The non-thermal processing technologies offer potential benefits over conventional methods.
• The crucial health benefits of minor millets have been demonstrated with examples from recent studies.
• Minor millets are beneficial in ensuring higher food security.

## 1 Introduction

The consumer demand for safe and nutritious food has increased in the past few decades. Consumers are in demand of food that is readily available, inexpensive and a good source of protein. (Sharma et al., 2023). In search of food with good protein content, the scientist came up with plant protein, and to be more specific, the proteins from millets. Millets are the oldest food grown in the semiarid region and can be grown with limited water availability. Millets are crops which can tolerate drought conditions; they belong to the Gramineae family of grasses (Pushpavalli, 2020). Millets are also referred to as the nutricereals due to the presence of a high proportion of proteins, dietary fibre, antioxidants, vitamins and minerals. Millet is a term that is commonly used for major millets and minor millets. Major millets include pearl millet, sorghum and finger millet, while minor millets include proso millet, kodo millet, foxtail millet, barnyard millet, brown top millet, etc. (Jacob et al., 2024). Unlike major millets, the minor millets were not so common among the urban population, and hence their demand was less (Pujari, & Hoskeri, J. H., 2022). However, due to an increase in awareness about the excellent nutritional profile of minor millets, their demand as a value-added product and as a food has gradually increased (Bhattacharya, 2023). In the present era, the minor millet not only provides food security but also justifies the health, nutrition, environment, and fodder at the minimum cost, thereby providing nutrition and food security opportunities (Chakraborty & Chakraborty, 2021).

Minor millets have served as the major source of nutrition for the population living in semiarid area. In India, the minor millets are an integral part of agricultural crops grown by the tribal population living in arid and semi-arid regions. The local population not only satisfies their hunger using the minor millets, but they also use these for ethnomedicinal purposes. The minor millets are now receiving escalating attention from food scientists and entrepreneurs, and hence, the research in this domain has accelerated to explore the utilisation of minor millets in the formulation of healthy food products. Minor millets are low glycemic index foods, contain biologically active components like phenolic compounds, they are gluten-free, making them a perfect food to fight the non-communicable disorders, which are on the rise due to a sedentary lifestyle across the globe (Kaur et al., 2023). The minor millets dietary fibre (15–20%) with a higher proportion of insoluble fibres, the protein content varies from 7 to 12%, with the highest protein content reported as 11.6, 12 and 12.3% in pearl millet, foxtail millet and barnyard millet, respectively. The minor millets are also a good source of essential amino acids like methionine, leucine, tryptophan, isoleucine, valine, threonine, etc. (Yousaf et al., 2021). Apart from the major food components, the minor millets are also a good source of B-complex vitamins like folic acid, B2, B3 and B1, minerals like iron, magnesium, zinc and calcium (Chauhan et al., 2018). The demand for food containing minor millet has increased in the recent past. The processing of the minor millet is essential to enhance the bioavailability of the nutrients, increase the palatability of food, and enhance the textural and sensory attributes of food. Numerous techniques are reported in the literature to process the minor millet into processed food products. The techniques range from the traditional household cooking, thermal techniques and emerging non-thermal processing. The processed millets usually show improved qualities. However, these are largely dependent on the processing method chosen. The present review gives a deep understanding of minor millets, their technological aspects, identifies the anti-nutritional factors in minor millets, focuses on the consequences of traditional and novel processing tools on the minor millets and gives details on the utilisation of minor millets in processed food products. This review will be of great importance to the technologists, scientists, and entrepreneurs exploring minor millets to meet the increasing demand from consumers.

## 2 Global production and consumption

The special qualities of millets, a broad category of small-grained cereals, are generating renewed interest. This review examines the current status of millet consumption and production worldwide, focusing on historical trends, regional variations, and the promising potential that these “nutricereals” offer. Africa and Sia emerge as the clear leaders in millet production. The Food and Agriculture Organization (FAO) states that Asia accounts for more than half (52.1%) of the world's total share, with India leading the way. Africa lags far behind in terms of both production and consumption. Millets play a crucial role in this diet, especially in the drylands, where their ability to withstand drought is extremely beneficial (Singh et al., 2020). Millets are a significant source of nutrition for people in countries such as Niger, Mali, and Burkina Faso, where they account for more than 40% of total cereal consumption (Anitha et al., 2023).

The consumption of millet has decreased globally in recent years, which is concerning given its historical significance. Studies indicate a reduction of approximately 1% (Agri, 2023). This can be linked to factors such as the increase in wheat and rice consumption, as well as the unfortunate perception that millets are only a food for the poor in certain areas. Still, a change for the better is happening. The United Nations' proclamation of 2023 as the International Year of Millets has served as a catalyst to increase consumer awareness and encourage the consumption of millets. This initiative has the potential to halt the decline and usher in a new era of appreciation for these nutritional powerhouses, especially given the unique nutritional profile of millets and the growing health consciousness.

**Fig. 1:**
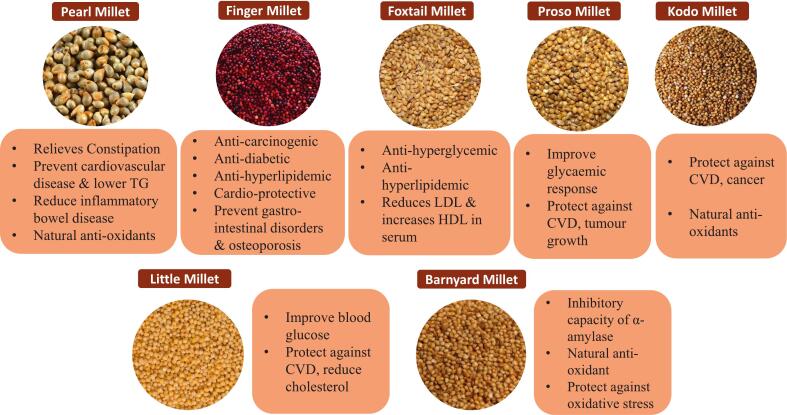
Therapeutic potential of minor millets.

Millets have a powerful nutritional punch. They have high concentrations of dietary fibre, protein, B vitamins, iron, and calcium, and are naturally gluten-free. Millets are a valuable addition to diets, especially in areas where malnutrition is a problem, as they often provide better sources of essential nutrients than wheat and rice. Furthermore, they have enormous potential for sustainable food production in a changing climate due to their hardiness in harsh environments and ability to flourish in marginal lands. Even with the bright future, difficulties still exist. The wider adoption of millets is still hampered by inadequate processing facilities, restricted market access for small-scale farmers, and low consumer awareness. To create novel value-added millet products, advance processing methods, and increase market viability, research and development initiatives are essential. Particularly in urban areas, educational campaigns that highlight the health advantages and adaptability of millets can help close the knowledge gap. Millets present a strong argument for a sustainable approach to achieving global food security. The global production and consumption of minor millets are described in Table 1. Through strategic investment in research and development, enhanced consumption patterns, and increased production, millets have the potential to become the golden seeds of a more secure and healthy future. (See Fig. 1.)

**Table 1:** Global Millet Production and Consumption (2023–2024).

**Country**	**Production 2023–24 (1000 MT)**	**Year on Year Change (%)**	**Global Share (%)**
India	12,200	−9.7%	39.7%
Niger	3400	−7.0%	11.0%
China	2700	0.0%	8.8%
Nigeria	2000	−1.5%	6.5%
Mali	1800	−1.8%	5.8%
Sudan	1600	−4.5%	5.2%
Ethiopia	1100	+16.8%	3.6%
Burkina Faso	1000	+10.1%	3.2%
Senegal	1000	−8.8%	3.2%
Chad	700	+0.9%	2.3%
Others	3302	+7.8%	10.7%
**Total**	**30,802**	**−4.1%**	**100%**

*Source: USDA data as reported by Mundus*Agri (2023).

## 3 Minor millets - poor man's meat

The term “poor man's meat” has been applied historically to minor millets, a diverse group that includes foxtail, kodo, and little millet (Singh et al., 2020). This reflects their accessibility and the lower-class population's use of them as a staple food. This moniker, though, undervalues their actual potential. With high levels of protein, dietary fibre, and vital minerals like calcium and iron, these little grains are incredibly nutritious (Anitha et al., 2023). This is comparable to, and often exceeds, the nutrient content of popular cereals such as wheat and rice. Minor millets are also drought-tolerant and resilient to harsh conditions, making them a promising solution to food security problems, especially in developing countries. Their adaptability provides a viable option for future farming methods. Minor millets can rightfully be recognized for their extraordinary nutritional value and adaptability, surpassing the historical label and making a positive impact on a world that is healthier and more food secure. A detailed comparison of the nutritional facts for all the minor millets is presented in Table 5.

### 3.1 Foxtail millet

The nutrient-dense, feathery-looking cereal known as foxtail millet is gaining popularity. This small seed, which belongs to the minor millet family, has many health advantages hidden beneath its humble appearance. Let's examine the nutritional makeup of foxtail millet and how it might fit into a balanced diet. Foxtail millet stands out for its protein content compared to other millets. Foxtail millet has a higher protein content than other minor millets, such as kodo millet and little millet, ranging from 5.9% to 8.3%. Additionally, this protein content is similar to that of some widely consumed cereals, such as wheat (*USDA National Nutrient Database for Standard Reference, Release,* 2015). Furthermore, foxtail millet protein is renowned for having a well-balanced amino acid profile that includes all eight essential amino acids, albeit in varying amounts (Yu et al., 2016) (Table 2). Although foxtail millet protein has certain limitations regarding the amount of certain essential amino acids, it can still be a valuable source, especially for vegetarian and vegan populations seeking plant-based protein options. Dietary fibre is essential for both general health and gut health. With both soluble and insoluble fibre fractions, foxtail millet is a good source of dietary fibre (Arora et al., 2023). The study also claims that the dietary fibre content of foxtail millet flour ranges from 8.9% to 10.6%. This fibre content encourages satiety, aids in maintaining regular digestion, and may even have advantages for controlling blood sugar levels (Ren et al., 2020). Beyond its protein and fibre content, the mineral content of foxtail millet is impressive (minerals like iron, calcium, magnesium, and phosphorus are abundant in it). Foxtail millet contains noteworthy amounts of both iron and calcium, which makes it a beneficial dietary option for people who may be deficient in these nutrients (Ahamed et al., 2013). This is especially important in areas where malnutrition and a lack of micronutrients are prevalent. In addition to its basic nutritional profile, foxtail millet may have other health advantages. Studies indicate that the presence of phenolic compounds may confer antioxidant properties. By helping the body prevent damage caused by free radicals, these antioxidants may enhance overall health and well-being. Furthermore, foxtail millet is naturally gluten-free, which means that people with celiac disease or gluten sensitivity can benefit from using it. The remarkable nutritional value of foxtail millet, particularly in terms of its protein, fibre, and mineral composition, makes it an invaluable addition to a balanced diet. Its potential health advantages, which include blood sugar regulation and gut health, reinforce its status as a superfood. With more studies being conducted to fully understand the potential of this tiny grain, foxtail millet is poised to become a common ingredient on plates worldwide, providing a tasty and nourishing base for a healthy lifestyle. (See Table 3.)

**Table 2:** Nutritional composition of selected minor millets (per 100 g) (ICMR – NIN 2020).

**Nutrient**	**Foxtail**	**Kodo**	**Barnyard**	**Little**	**Proso**
**Protein (g)**	11.2	8.3	11.2	7.7	12.5
**Dietary Fibre (g)**	8.0	9.0	10.1	7.6	7.0
**Iron (mg)**	2.8	0.5	15.2	9.3	0.8
**Calcium (mg)**	31	27	11	17	14
**Magnesium (mg)**	81	92	68	114	106
**Phosphorous (mg)**	290	188	280	220	206
**Gluten Free**	Yes	Yes	Yes	Yes	Yes

**Table 3:** Effect of thermal and non-thermal techniques on characteristics of minor millets.

**Millet**	**Method**	**Objective**	**Conditions of the applied method**	**Results**	**References**
Sorghum	Microwave	To stabilise the product	350 W to 500 W for 45 s	Decrease in acidity of fat, viscosity and anisidine value. Enhanced range of disrupted starch	(Li et al., 2021)
Pearl millet	Microwave	To stabilise the product	900 W for 100 s	Lipase activity was reduced by 91.2%.	(Sundaresan et al., 2025)
Proso millet	Microwave	Enhancement of physical and chemical parameters	600 W for 6 to 30 min	Protein content was increased	Bangar et al., 2022).
Pearl millet	Cold plasma	Change in functional characteristics	40 to 45 kV for 10 to 15 min	Swelling capacity, solubility and water absorption capacity were increased. Components such as fibre, protein and moisture content were reduced.	(Sarkar et al.,2023)
Little millet	Cold plasma	To modify the functional parameters	10 to 25 kV for 20 to 30 min	Oil absorption capacity and water absorption capacity were elevated. Viscosity and colour of cooked paste were reduced.	(Sun et al., 2022)
Pearl millet	Ohmic heating	Change in cooking parameters	Frequency required: 60 Hz	Less yield, as well as a decrease in solubility and absorption parameters.	(Dias et al., 2019)
Sorghum	infrared	To stabilise the product	120 °C for 8.5 min	An 80% decrease in free fatty acids, moisture and viscosity was observed.	(Semwal and Meera, 2023).
Sorghum	irradiation	Removal of contaminants	Dose: 10, 25 kGy	Microbial activity was reduced by 3 log cycles. Amylase activity, both alpha and beta, was decreased	(Liang et al., 2024)

### 3.2 Kodo millet

Kodo millet is a small, round grain that is part of the minor millet family. It is gaining recognition due to its remarkable nutritional profile. Kodo millet is sometimes overlooked in favour of its more well-known relatives, but it has a surprisingly high nutritional content. Using the most recent research as a guide, let's examine the distinct nutritional makeup of kodo millet and consider any potential health advantages. Kodo millet is a noteworthy choice for plant-based protein sources for vegetarians and vegans due to its high protein content. According to recent research by Meena et al. (2020), the protein content of kodo millet ranges from 6.8% to 8.9%, which is higher than that of other frequently consumed cereals, such as rice. Comparing this protein content to other minor millets, such as little millet, is also noteworthy (Joshi et al., 2013). All eight essential amino acids are also present in kodo millet protein, although some of the amounts may be lower than the required levels (Ahamed et al., 2013). To create a complete protein profile, this can be lessened by combining kodo millet with other complementary protein sources, such as legumes.

Kodo millet excels in providing dietary fibre, which is an essential part of a balanced diet. Kodo millet flour has a high dietary fibre content, ranging from 10.6% to 13.8% (Table 2). Because it slows down the absorption of carbohydrates, this fibre content helps maintain regular digestion, increases feelings of fullness, and may even have some beneficial effects on blood sugar regulation. Furthermore, Kodo millet's soluble and insoluble fibre fractions offer a comprehensive benefit to digestive health. Kodo millet provides an impressive range of vital minerals, in addition to protein and fibre. Minerals, including iron, calcium, magnesium, and phosphorus, are abundant in it (Hadimani et al., 2020). Kodo millet contains significant amounts of calcium and iron, making it a beneficial dietary option for individuals at risk of deficiencies, particularly in areas where malnutrition is prevalent (Hadimani et al., 2020). The mineral profile of kodo millet makes it a viable dietary intervention for addressing micronutrient deficiencies, particularly iron deficiency anaemia, which remains a significant global health issue. Kodo millet may offer additional health benefits beyond its nutritional profile. Furthermore, the antioxidants present in kodo millet can support the body's defense against damage caused by free radicals. Kodo millet is a valuable addition to a balanced diet due to its impressive nutritional content, particularly its rich mineral composition, fibre, and protein. Its potential health advantages, which include better digestion and blood sugar regulation, reinforce its status as a superfood. Kodo millet is a versatile grain that can be incorporated into a variety of culinary creations due to its distinct qualities and ease of digestion. Kodo millet is set to gain wider recognition and appreciation as a delicious and nutritious staple grain, as research into its full potential continues.

### 3.3 Barnyard millet

A gluten-free grain with an unusually elongated shape, barnyard millet (*Echinochloa* spp.) is becoming more well-known for its outstanding nutritional profile. Barnyard millet is often overlooked in favour of its more popular counterparts, but it remains a valuable addition to a diet rich in nutrients. Using the most recent research as a guide, let's examine the rich nutritional makeup of barnyard millet and consider any potential health benefits. One notable feature of barnyard millet is its high protein content, which can range from 6% to 13% (Ranganathan et al., 2020). This is greater than other staple grains, such as rice and wheat (*USDA National Nutrient Database for Standard Reference, Release,* 2015). Among other millets, barnyard millet protein is notable for having a reasonably well-balanced amino acid profile (Ahamed et al., 2019). Although the amount of some essential amino acids is limited, barnyard millet provides a solid base for plant-based protein consumption. Dietary fibre is crucial for maintaining healthy digestion and overall well-being. With a total dietary fibre content ranging from 9.5% to 14%, barnyard millet is a good source of both soluble and insoluble fibre fractions (Table 2) (Chandrasekhara et al., 2016). Because it slows down the absorption of carbohydrates, this fibre content helps maintain regular digestion, increases feelings of fullness, and may even have some benefits for blood sugar regulation (Yu et al., 2016). Due to its abundance in vital minerals, barnyard millet is a highly recommended dietary option for people who may be deficient in certain minerals. It has higher iron content than other cereals such as wheat and rice (Ranganathan et al., 2020). Furthermore, other important minerals like calcium, phosphorus, and magnesium are also present in barnyard millet (Ahamed et al., 2019). Barnyard millet's mineral profile makes it a viable dietary option for addressing micronutrient deficiencies, particularly in areas where malnutrition is prevalent.

Barnyard millet is a valuable addition to a healthy diet due to its impressive nutritional content, particularly its rich mineral composition, fibre, and protein. Its potential health advantages, which include helping with blood sugar regulation and digestion, reinforce its status as a superfood. It's also safe for people with celiac disease or gluten sensitivity because it's gluten-free. Barnyard millet is a small grain that could become a staple on plates worldwide as research into its full potential continues. It provides a tasty and nutritious base for a healthy lifestyle.

### 3.4 Little millet

Little millet (*Panicum sumatrense*), a neglected nutraceutical grain, is gaining popularity due to its impressive nutritional profile. Little millet, which is frequently overlooked in favour of its larger millet counterparts, is a valuable addition to a healthy diet because it provides an unexpected array of nutrients. Using the most recent research as a guide, let's examine the distinct nutritional makeup of little millet and its potential health benefits. Little millet is a promising source of plant-based protein. The protein content of little millet flour, ranging from 6.3% to 8.4%, is reported by Dey, Kumar, and Saxena (2022), although it can vary depending on the growing conditions. This protein content is similar to other minor millets and exceeds that of some commonly consumed cereals, such as rice. Furthermore, in comparison to other cereals, millet protein has a relatively balanced composition of essential amino acids. Nevertheless, there are restrictions on the quantity of some essential amino acids; therefore, a complete protein profile requires diet diversification or the combination with complementary protein sources. Dietary fibre is crucial for maintaining healthy digestion and overall well-being. In this regard, little millet stands out as a champion. At 18.4% to 21.7%, little millet flour has a high dietary fibre content (Table 2), as reported by Prabhavathi et al. (2019). Little millet boasts an impressive mineral profile, making it an excellent dietary choice for individuals at risk of mineral deficiency. Little millet is also a good source of calcium, magnesium, iron, and other important minerals. Little millet is positioned as a viable dietary intervention to address micronutrient deficiencies due to its mineral-rich profile, especially in areas where malnutrition is prevalent. Little millet is a valuable addition to a healthy diet due to its exceptional nutritional content, particularly its rich mineral composition, fibre, and protein. Its potential health advantages, which include better digestion and blood sugar regulation, reinforce its status as a superfood. The full potential of little millet as a dietary staple can be realized through more investigation into its functional qualities and health advantages. The appeal of little millet in promoting healthy eating habits worldwide is enhanced by its ease of incorporation into a variety of culinary creations.

### 3.5 Proso millet

The remarkable nutritional profile of proso millet (*Panicum miliaceum*), which is frequently overlooked in favour of birdseed, is becoming more widely known. This small grain is a great complement to a balanced diet because it is packed with vital nutrients. Using the most recent research as a guide, let's examine the rich nutritional makeup of proso millet and consider any potential health benefits. Proso millet is notable for having a protein content that ranges from 8.3% to 14.0%. This is greater than other staple grains, such as rice and wheat. Among other cereals, proso millet protein is noteworthy for providing a reasonably well-balanced amino acid profile (Ahamed et al., 2018). Proso millet is a good starting point for plant-based protein intake, especially when combined with complementary protein sources, although there are certain limitations regarding the amount of essential amino acid lysine it contains. A good source of soluble and insoluble fibre fractions is proso millet. The fibre content helps maintain regular digestion, increases feelings of fullness, and may even have some benefits for blood sugar regulation (Yu et al., 2016). Proso millet has notable manganese content, even higher than that of commonly consumed whole grains such as whole wheat (Hadimani et al., 2016). Proso millet is also a good source of iron, magnesium, and phosphorus, among other important minerals (Table 2) (Ahamed et al., 2018). Due to its mineral composition, proso millet can be utilized as a dietary supplement to address micronutrient deficiencies, particularly in regions where malnutrition is prevalent. A nutritious diet can greatly benefit from the inclusion of proso millet due to its remarkable nutritional content, particularly in terms of its protein, fibre, and mineral composition. Its potential health advantages, which include helping with blood sugar regulation and digestion, reinforce its status as a superfood. It's also safe for people with celiac disease or gluten sensitivity because it's gluten-free. With more research being conducted to fully understand proso millet's potential, this underappreciated grain has the potential to become a global staple on plates, providing a tasty and nourishing base for a healthy lifestyle.

## 4 Traditional use of minor millets

Millets are known for being a rich source of various nutritious components, bioactive compounds, and have the potential to serve as a major source of nutrients for millions of people in the world (Gaikwad et al., 2021). In the current global scenario, the most prominently grown minor millets are pearl millet (*Pennisetum glaucum*), foxtail millet (*Setaria italica*), proso millet (*Panicum miliaceum*), finger millet (*Eleusine coracana*), kodo millet (*Paspalum scrobiculatum*), little millet (*Panicum sumatrense*), and barnyard millet (*Echinochloa colona*). Minor millets are capable of serving as a source of food and nutritional security for vulnerable sections of the population. They have also traditionally played a significant role in farming and food culture in various parts of the world, mainly Sub-Saharan Africa and South Asia. India is recognized for its large-scale production of minor millets globally (Padulosi et al., 2015). Traditional millet-based foods and beverages are prepared and consumed in various parts of Africa, Asia, and the Indian sub-continent. The most significant use of millet-based foods includes whole-grain foods, flour-based foods (flatbread, dumplings, porridges, etc) and beverages of alcoholic and non-alcoholic variety. Pearl millet, foxtail millet, kodo millet, and little millet are consumed as whole grains, similar to cooked rice products. Some of the important methods, like popping and germination, are traditionally used for consumption in various parts of the world. The popping process usually moistens the grains, removing the outer pericarp, and further, the grains can be consumed as snacks or utilized for milling. Germination aids in increasing the protein, minerals, and vitamins content, and the seeds can be consumed either raw or cooked. Traditionally, germinated grains were considered for infants and elderly people (Paschapur et al., 2021). The foods prepared from millet flour vary in different geographic regions of the world. Various food items are prepared and consumed by the tribal community in India. Chapatis have been prepared by baking pearl millet flour in the mostly southern part of India. A very popular sweet porridge is made from barnyard millet, which is famous in Uttarakhand, India (Verma et al., 2018). A tribal community in Kerala (India) is known as the Muthuvan. They consume a local food preparation, ‘katty’, which is a type of pudding that is prepared with finger millets (Rawat et al., 2021). In countries like Africa, food formulation prepared from millet, named ‘ogi’, is consumed as a staple food. Millet-based fermented products, such as ‘porridge’, are also included in the meals of infants (Adebiyi et al., 2018). Traditionally, pearl millet is considered for stimulating milk production among the Northern Benin group of people. Energy-rich food formulations prepared from malted millets, such as ragi and sorghum, can be utilized as weaning foods and have the potential to eradicate malnutrition in various underdeveloped and developing countries. Millets are considered to have notable traditional roles. Therapeutic activity of proso millet, barnyard millet, and kodo millet is considered for gonorrhoea, spleen disease, and conventional constipation conditions (Jacob et al., 2024). The roles of pearl millet are known for its appetising effect, foxtail millet for its modulatory role in sleep and treating constipation, and kodo millet for purifying blood, among others. Finger millets are traditionally recognized as suitable for diabetic patients due to their low glycaemic index (Nakarani et al., 2021). Traditional application of millets is extended to formulate alcoholic and non-alcoholic beverages in many countries like Africa, East Asia and India (Amadou, 2019). Currently, the detrimental impact of global climate change has led to a crisis of nutritionally rich crops in specific regions, resulting in poor nutrition. Millets are poor man's crops and can withstand environmental stresses. They are ideal for sustaining global food security, stability in yields and thus can ensure health and nutrition in the challenging areas of the world (Saleem et al., 2023).

## 5 Consequences of processing on minor millets

### 5.1 Consequences of thermal processing on minor millets

#### 5.1.1 Ohmic heating

Ohmic heating is the method of thermal processing in which an electric current is passed through a conductor to increase the temperature. The thermal rise inside the material is raised volumetrically at a very fast pace (Kabui & Athmaselvi, 2024). It has been reported that ohmic heating is considered a feasible technique for processing of minor millets such as Foxtail millet and proso millet. The important characteristics of this method are that it is very efficient, consumes low energy, possesses a very short processing time, and is recyclable. The heating rate and uniformity of this method are due to the combination of electric conductivity and electric field of the millet being processed (Dias-Martins et al., 2019). During ohmic treatment, there is a rise in temperature which results in migration of moisture, denaturation of protein and gelatinisation (Llave et al., 2018). Studies reported that yield of kodo millet is low after ohmic heating treatment 50 °C for 2.5 h is given as compared to normal traditional cooking techniques, during thus study several properties were investigated such as yield, colour, water activity and solubility activity (Joshi et al., 2023; Kabui & Athmaselvi, 2024) The softening rates of rice grains are higher in ohmic heating than conventional heating methods, because heat given is distributed evenly throughout the mixture. Therefore, at the same time, gelatinisation of starch occurs. It has been proven that ohmic heating has a higher rate of starch gelatinization compared to other cooking methods (Chen et al., 2024). The microstructure of the minor grains is observed to be more porous compared to normal cooked grains; this is attributed to the structural change caused by electroporation during ohmic heat treatment.

#### 5.1.2 Microwave processing

Dipole rotation and polarization of ions is employed as a basic principle of microwave processing, as well as electromagnetic radiation (EMR) is applied between the wavelengths of 0.3–300 GHz. During microwave processing, some of the energy applied is absorbed, while a portion is transmitted and reflected. The interactions between microwaves depend on both the dielectric loss and the dielectric constant. The anti-nutritional compounds present in several millets and their products are reduced when treated with microwave processing (Latha Ravi & Rana, 2024). This reduction in antinutritional components is attributed to the breakdown of peptide bonds, covalent bond separation, and disintegration of disulfide bonds. The concentration of inositol and its phosphate is decreased by the production of free radicals during the microwave processing treatment. The denaturation of heat-treated proteins helps in the deactivation of trypsin inhibitors. It has been reported that tannins present in millets are decreased when treated with 900 W for 30- 90s, particularly in sorghum, finger, and pearl millet by 76.53%, 42.52% and 44.18% (Suhag et al., 2021). There are several grain processing methods, such as drying, enzyme inactivation, sterilization, and disinfection operations, for which microwave technology is utilized. Several functional and biochemical aspects, as well as their impact on minor millets, were studied. It was found that there is a rise in TPC from 2.74 to 4.57 mg GAE/100 g, as well as a reduction in antioxidant activity from 90.12% to 87.87%, when the power and duration levels of the microwave are elevated (Wilson et al., 2022). According to Tagade and Sawarkar (2023), the capacity for oil absorption is reduced in foxtail millet flour, whereas the capacity for water absorption is enhanced after microwave treatment. It has also been reported that the swelling power of millet flour is reduced when the power and time of microwave processing are increased. Stabilization of sorghum flour is achieved through microwave treatment, which reduces the acidity of flour lipids during storage. The lipase is partially inactivated, and oxidation of free fatty acids is reduced by microwave processing (Adebowale et al., 2020). The starch present in millets can be damaged due to the thermal effects of microwave heating, as it interferes with the molecular interactions of starch and proteins; therefore, the viscosity is also reduced. The sensory properties of several millet-based products were also studied, and no negative effects were observed on their sensory attributes (Lazim & Nasur, 2017). It has also been reported that the formation of hot spots results from microwave processing due to the non-uniformity of temperature distribution. The germination rates of grains are reduced after the generation of these hot spots. Another study showed that the lipase activity of pearl millet is markedly decreased by microwave heating (Sruthi & Rao, 2021). During storage of flour, there is an increase in rancidity; however, this was significantly lower in microwave-processed products.

#### 5.1.3 Infrared processing

Infrared radiation is another processing technique in which the range lies between the wavelength of microwave and visible light. There are commonly three regions of wavelengths which are: first IR (0.78–1.4 m), second IR (1.4–3.0 m) and third IR which lies between 3.0 and 1000 m. The heat is generated as a result of vibration caused in the water molecules after the application of infrared radiation (Simeone et al., 2024). There are several benefits of infrared processing, including decreased heating time, homogeneous heating, low energy consumption, maximum heat transfer rate, and enhanced product quality. After the application of infrared radiation, heat energy is produced, which can cause disruption in microbial cells, separation of mesosomes, and contraction and leakage in cytoplasmic membranes and intracellular compounds, respectively (Kumari et al., 2025). Inactivation of lipase is caused by the breakdown of lipids after the infrared heating, which also leads to a reduction in the production of free fatty acids. Studies have shown that the content of free fatty acids decreases with an increase in temperature and time of infrared radiation processing. During infrared radiation treatment, it was observed that lipase activity is decreased by 87.88% in sorghum flour at 120 °C for 10 min, and a marked reduction in moisture was also observed in different products. Elevation in protein, fat, and fibre was observed after the reduction in moisture (Dey et al., 2024). Due to the denaturation of proteins observed during this thermal treatment, a reduction in protein digestibility, from 6.7% to 22.3%, has been noted. The starch digestibility of grains is significantly increased, attributed to gelatinization when treated with the infrared heating method (Simeone et al., 2024). This method also decreased the anti-nutritional compounds, such as phytic acid and tannins, as well as several phenolic compounds, in sorghum grains.

#### 5.1.4 Radio frequency processing

The non-ionizing EMR waves within the range of 1 to 300 MHz are called radio frequency waves. These waves can enter dielectric materials via ionic polarization as well as dipole rotation, which leads to the production of heat volumetrically. Radiofrequency waves penetrate more deeply into a product as compared to microwaves due to their shorter wavelength. It has been observed that these wavelengths are utilized for several processes, such as the inactivation of microbes and enzymes, modification of proteins, and drying and roasting of grains like rice, sorghum, foxtail, pearl, and maize (Chen et al., 2024). The primary disadvantage of this processing technique is that it does not generate uniform heat due to the uneven distribution of radiation between the sample and its environment. It has been studied that the performance and efficiency of this method can be enhanced when combined with hot air, which improves the uniform distribution of heat (Kaur & Kaur, 2024). The functional and other physicochemical properties of pearl millet were investigated after treatment with combined hot air and radio frequency technology. The results showed that the water activity capacity of pearl millets was increased as compared to raw pearl millet. These higher water activity capacity values will enhance the processing parameters of pastes and dough (Yarrakula et al., 2022). It was also found that this method raised the solubility of proteins, antioxidant activity, and total phenolic content of the treated samples (Sunil et al., 2024). Other properties, such as foaming capacity, emulsification, and colour, were also improved markedly.

### 5.2 Consequences of non-thermal processing

#### 5.2.1 Ultrasound processing

Ultrasound is one of the leading methods in non-thermal treatments, providing several benefits with minimal processing, enhanced safety, and improved quality of the food product. The frequency range of ultrasound waves lies between 20 kHz and 100 kHz. These waves have high energy when transmitted through any sample. A sequence of rarefaction and compression cycles is created, which causes disturbance between the molecules, and modification of the food sample occurs (Mounika et al., 2024). The inactivation of enzymes, formation of micro channels, and development of free radicals occur during the destruction of cell membranes, which is caused by the cavitation process. The cavitation process is the mechanism by which the energy required is transmitted into the food matrix; thus, the process causes the development, expansion, and disruption of gas bubbles (Mudau & Adebo, 2025). The emulsifying properties of food proteins are improved after ultrasound treatment. The effect of ultrasound technique on the functional and structural properties of millet proteins was studied by Thakur et al. (2023). This study revealed that the solubility of millet protein concentrate is higher after being treated with ultrasound, as the hydrophilic groups of amino acids are exposed to water. The digestibility of proteins is increased by 13.3% while the foaming ability is reduced. The swelling properties of granules are decreased by the induction of cavitation and oscillation during the processing operation, which facilitates the easy penetration of water and reduces viscosity (Pandey & Singh, 2024). The swelling power of the starch increases when exposed to ultrasound processing for 25 min, with an increase in both the concentration of the slurry and the exposure time. The water absorption capacity is increased, which in turn improves the binding between water molecules and protein chains, such as amylose and amylopectin. There is a direct relationship between the yield of polyphenols; these components are released after molecular collisions facilitated by the ultrasound technique (Makwana et al., 2025). In the case of pearl millet, approximately 30% of the yield was increased when treated with ultrasound at 250 to 500 W. It was found that the content of anti-nutrient compounds present in finger millets is reduced after being assisted with ultrasound processing treatments. The hydration properties of grains are improved when the amplitude of ultrasound waves is higher, which disrupts the cells and results in the formation of micro cavities, thereby elevating mass transfer via diffusion (Alkaltham et al., 2024). It was also found that the total phenolic count of finger millets decreased with an increase in amplitude and time of ultrasound, which led to the formation of cavities in the cellular structure, thereby increasing the permeation of solvents (Prakash et al., 2024). This technique facilitates the conversion of tannic acid to gallic acid, resulting in a reduction of tannins in finger millets.

#### 5.2.2 Cold plasma

The cold plasma method is another non-thermal technique performed at normal room temperature, which is why it is known as cold treatment. This method has no negative impact on the surroundings, as it does not produce chemicals or toxins in the treated sample (Jaddu et al., 2024). Among all the other techniques, cold plasma can be developed at low atmospheric pressure, followed by an airflow rate of 0.01 m^3^/h. It was found that the quantity of anti-nutrient (phytic acid) decreased from 60.64% to 39.23% when stored for 1 to 2 h under cold plasma processing conditions. They also revealed that combining cold plasma with the normal soaking process reduces the concentration of phytic acid to 21.2% in pearl millets and wheat grains (Balendran et al., 2024). During cold plasma treatment, the crystallinity of finger millet is decreased due to the starch depolymerisation. This treatment also disrupts the microstructure of starch granules, as well as the properties of minor millets, including water absorption capacity, solubility, viscosity, and swelling capacity. Oil retention capacity is increased due to the presence of nonpolar amino acids and other protein structures in millets (Sonkar et al., 2023). Another study was conducted on proso millet starch, examining the effect of cold plasma treatment combined with ultra-high pressure on physical, chemical, and digestive parameters. The results showed that slight changes were observed on the surface of the starch granules caused by non-penetrative disruption of plasma etching (Sarkar et al., 2023). The colour intensity of porso millets was reduced when the time of treatment was increased. This show that cold plasma treatment raised the movement of dual helices and results in the loss of radial orientation that leads to the poor refraction (Lokeswari et al., 2021) The viscosity of starch of proso millet was reduced in cold plasma treated samples, that is attributed to the disruption of polymers present starch and thus leads to the changes in structure (Charu et al., 2024). The internal structure of starch granules may be altered due to the long exposure time to cold plasma treatment. This prolonged exposure elevates the enzyme-interacting sites and increases the susceptibility of starch to enzymatic digestion.

#### 5.2.3 Pulse electric field

The utilisation of short pulses of electric fields with maximum intensities ranging from 10 to 80 kV/cm is referred to as the pulse electric field. It is applied to food materials placed between two electrodes for a few microseconds. The shelf life is increased due to enzyme inactivation as well as the inhibition of pathogens, which helps maintain the overall quality of food materials, including grains, in terms of both nutritional and sensory parameters (Kang et al., 2024). The hydration properties and isolation of bioactive components, as well as the extraction of oils from grains and millets, can be enhanced with the assistance of a pulse electric field. Zhang et al. (2023) investigated the impact of a pulse electric field with an intensity of 2 kV/cm on sorghum, revealing that the antioxidant content (32.9%) and total phenolic count (25.3%) were higher in the treated samples. The porosity of the cell membranes of sorghum and finger millets was improved due to electroporation. The disruption of starch granules was also found to be higher in the pulse electric field, resulting in an increased surface area of the granules; therefore, more phenolic compounds are released, which are found within the matrix of protein and carbohydrate (Ramakrishnan et al., 2023). Studies have revealed that pulse electric field can be utilized effectively as an initial treatment prior to drying of starch paste of millets. The drying time was also significantly reduced in red rice starch paste after treatment at intensities of 10 and 30 kV/cm. The pulse electric field technique has also shown a decrease in the enthalpy and gelatinization temperature of millet and grain starch, such as porridge and finger millet, as well as wheat (Kheto et al., 2024). This reduction in enthalpy is due to the penetration of water molecules into the crystalline structure of starch, resulting in a disrupted crystalline structure. This reduction in crystallinity causes a fall in gelatinisation temperature.

#### 5.2.4 High-pressure processing (HPP)

High-pressure processing is a technique that utilizes uniform pressure throughout the entire food product, regardless of its size and shape. The pressure used ranges from 100 to 800 MPa at standard room conditions. This technique can be applied to both types of foods, including solid and liquid products, regardless of whether they are packed or not, and it effectively kills microbes (Ramakrishnan et al., 2023). A study observed that the digestibility of pepsin was lower in proso millet when treated with high-pressure processing at 200 to 600 MPa for 10 to 20 min. The biochemical and morphological parameters of millet protein glaidin were also observed, and the reduced surface hydrophilicity of glaidin was revealed due to the modifications in the structure of the protein. The distribution of protein is caused by the development of protein agglomeration, which leads to an increase in the size of the protein particle (Bangar et al., 2022). It was also found that the solubility and enthalpy of protein gluten are raised as the pressure during the process increases. Inactivation of microbial species, such as yeast and Lactobacillus, present in foxtail millet occurs when treated with high-pressure processing at 400 MPa for 10 min at ambient conditions. However, complete destruction of microbes was achieved at 400 MPa for 60 min at 65 °C and 600 MPa for 10 min at 66 °C. A reduction in glucoamylase and α-amylase activity to 20.4% and 58%, respectively, was also observed at a 400 MPa treatment for 10 min at 65 °C in the case of glaidin and porso millet (Chandrasekara & Shahidi, 2022). The degree of gelatinisation was improved and enhanced from 0.41 to 63.93% in germinated foxtail millets (Sharma et al., 2018). The diffusion coefficient was found to be higher in germinated foxtail grains (6.76 × 10^−9^ m^2^/s at 200 MPa at 60 °C) as compared to the non-germinated foxtail grains (5.66 × 10^−9^ m^2^/s at 400 MPa at 80 °C) (Kang et al., 2024).

#### 5.2.5 Food irradiation

It is another non-thermal treatment that utilizes the energy efficiently, and it is used particularly for the preservation of food items. This process involves exposing the food product to ionizing and non-ionizing radiations, which may include UV, infrared, visible, and radio waves, as well as X-rays, gamma rays, and accelerated electron beams. These radiations directly affect carbohydrates, fats, and other nutrients, while some interact with food components indirectly (Francis et al., 2022). The potential of the food irradiation technique was utilized for the sterilization and extension of the shelf life of minor millets, such as foxtail and porso millet, and the quality of these millets was also assessed. It was observed that this technique reduces the lipase content in foxtail millet, which is attributed to a decrease in free fatty acids, thereby increasing shelf life and stability (Manjula et al., 2015). The anti-nutritional components, such as phytic acid, present in sorghum were reduced after irradiation at different doses of 10, 15, 20, 25, and 30 kGy, and the reduction values were 39%, 49%, 66%, 79%, and 90%, respectively. This reduction in phytic acid is caused by the degradation of the chemical structure of phytate. Another parameter, such as millet composition and digestibility, was also studied, and the digestibility of protein present in sorghum was also increased (Sujatha et al., 2017). The moisture content of finger millet decreased after irradiation processing; however, this treatment resulted in an increase in catalase, superoxide dismutase, and radical scavenging activity, as well as a reduction in lipoxygenase activity (Huang et al., 2022). Irradiation causes the degradation of amylose and amylopectin in starch, which leads to the reduction of the viscosity of starch; therefore, the water-binding capacity of starch will be reduced, which decreases the swelling ability of millet granules.

## 6 Anti-nutritional components in minor millets

Anti-nutritional compounds are natural organic elements that are an integral constituent of foods, hindering the absorption and bioavailability of various minerals, vitamins, proteins, and carbohydrates. Some of the well-known anti-nutritional components include phytates, oxalates, tannins, and inhibitors of trypsin or protease (Dey, Saxena, et al., 2022). Millets are reported to contain anti-nutritional factors, and most of the studies showed that tannins and phytates are mainly present in millet grains. They are also reported to contain trypsin-inhibitory factors and phenolic compounds. The phytate content was found to be higher in proso and foxtail millets. Tannin concentration was reported to be highest in kodo millet, followed by finger and barnyard millet grain (Sharma & Gujral, 2019). The trypsin inhibitors were present in barnyard and finger millet grains in the range between 7.85 and 8.36% (Gull et al., 2016). Phytic acid, also known as phytates, is capable of binding to proteins, leading to subsequent precipitation and reducing their bioavailability, as well as lowering the activity of various enzymes. They are also reported to hinder the absorption of minerals, namely calcium, iron, zinc, and magnesium, as a consequence of phytate-metal insoluble complex formations. Studies have found that tannins also form insoluble complexes with proteins and minerals, thereby causing mineral deficiency and digestive disorders (Raes et al., 2014). Trypsin inhibitors mainly impact the metabolism of sulphur, amino acids, and decrease protein digestibility due to ineffective utilisation of amino acids (Sharma & Gujral, 2019).

It is crucial to look for processing technologies that will break down phytic acid, tannins and other anti-nutritional factors present in minor millets. Studies have reported effective food processing practices that improve the nutritional value and bioavailability of micronutrients, while also reducing anti-nutritional factors (Kushwaha et al., 2019). The application of conventional mechanical processing, such as decortication, milling, and sieving, to millet grains is beneficial in reducing the content of anti-nutrients; however, it may also significantly impact the nutritional value. There is an urgent requirement to develop advanced technologies to increase the yield of nutrient-dense minor millet flours (Dey, Kumar, & Saxena, 2022). Bioprocess technologies, such as germination, soaking, fermentation, and enzymatic hydrolysis, are reported to enhance the nutritional content while efficiently reducing antinutrients in minor millet grains. Germination of minor millets showed effective reduction in anti-nutritional factors, specifically phytic acids, tannins, and polyphenols (Bhavadharani & Gurumoorthi, 2025). This technique was also reported to enhance the bioavailability of certain micronutrients, such as calcium, zinc, and iron, in minor millets. The soaking method is considered one of the ancient techniques resulting in a decrease in anti-nutrients in minor millets. Unlike soaking, the sprouting of minor millets resulted in a reduction of anti-nutritional compounds, such as phytic acid and tannins. Fermentation technology has been reported as an effective method for reducing trypsin and amylase inhibitors, as well as phytic acids, in millet grains (Boakye et al., 2023).

## 7 Utilisation of minor millets in processed food

The crucial health benefits of minor millets have been demonstrated in various studies by the research community. Significant evidence demonstrated the preventive properties of millets in lifestyle ailments and age-related disorders. The medical properties of millets are mainly due to the presence of various bioactive components like polyphenols, flavonoids, essential fatty acids, amino acids, vitamins, and minerals in millet grains. Minor millets were considered functional foods due to their crucial health benefits beyond their nutritional capacity. They were reported to contain resistant starch, functional lipids, phytosterols, lignans, and oligosaccharides, and these nutraceuticals were capable of preventing cardiovascular diseases, metabolic syndrome, cancer, and gastrointestinal diseases (Balakrishnan & Schneider, 2022).

The application of minor millets in food product development has been practiced from ancient times, utilising them in the traditional preparation of foods. However, the superior functional and nutraceutical adequacy of minor millets make them ideal for the development of non-conventional processed foods. Minor millets were utilized to develop gluten-free pasta products, as millets are naturally devoid of gluten protein (Jnawali et al., 2016). Studies evaluated the nutritional and functional properties of gluten-free pasta made from minor millet grains and showed excellent results from both biological and technological standpoints (Cordelino et al., 2019). The functionality of gluten-free products developed with minor millets was enhanced through the application of hydrocolloids and hydrothermal processing technology (Gao et al., 2018; Rudra et al., 2020). Minor millets have been widely utilized in the formulation of bakery products, muffins, breads, and extruded products, yielding acceptable functional and sensorial properties. Some of the processed food products, utilising minor millets, as reported by various researchers, are summarised in Table 4.

**Table 4:** Processed food products developed by utilising minor millets.

**Processing Technology**	**Minor Millet Utilized**	**Developed Food Product**	**Important Findings**	**Reference**
**Flaking and Popping**	Finger Millet	Flakes	i) Desirable bulk density, hardness and sphericity of decorticated expanded millet flakes. ii) Highly cost-effective and nutritious.	Sircar et al., 2019
	Pearl Millet	Flakes	i) Good water absorption capacity, swelling capacity and dispersibility of the flakes ii) Rich in minerals, protein and fibre content.	Adebanjo et al., 2020
	Finger Millet	Popped Millets	Popping increased the bioaccessibility of iron in the products.	Krishnan et al., 2012
**Baking**	Finger Millets	Biscuits	i) Rich in iron ii) Gluten-free with good overall acceptability iii) Up to 50% substitution with finger millets was acceptable.	Singh & Srivastava, 2008; Kaur et al., 2020
		Bread	Improved dough stability and extensibility with hydrothermally treated finger millets.	Onyango et al., 2020
		Muffins	Improved nutritional quality and gluten-free.	Jyotsna et al., 2016
	Pear Millet	Cookies, biscuits and cakes	Replacement of wheat flour up to 60% with pear millet flour resulted in acceptable organoleptic quality.	Kulkarni et al., 2021
**Extruded products**	Pear millets, Finger millets and others	Macaroni, spaghetti	Improved nutritional quality (crude fibre and protein-rich); desirable functional properties; appropriate for weaning foods, snacks for adults.	Sukumar & Athmaselvi, 2019; Rolandelli et al., 2020
**Beverages**	All the minor millets	Alcoholic and non-alcoholic beverages	Traditionally used in various countries like Namibia, Nigeria, Uganda, Kenya, Taiwan, India, Nepal, and Bhutan.	Saleem et al., 2023

**Table 5:** Nutritional facts for all five millets per 100 g (uncooked), with values and %DV based on FDA's 2024 Daily Values (2000 kcal diet).

**Nutrient**	**Foxtail Millet**	**Kodo Millet**	**Barnyard Millet**	**Little Millet**	**Proso Millet**
**Calories (kcal)**	351	353	300	329	356
**Protein (g)**	7.3 (15%)	7.7 (15%)	9.8 (20%)	7.7 (15%)	11.0 (22%)
**Total Fat (g)**	4.3 (6%)	3.6 (5%)	3.9 (5%)	4.7 (6%)	4.0 (5%)
**Total Carbohydrate (g)**	63.2 (23%)	65.9 (24%)	55.0 (20%)	67.0 (24%)	70.0 (25%)
**Dietary Fibre (g)**	9.5 (34%)	12.0 (43%)	12.5 (45%)	20.0 (71%)	8.5 (30%)
**Iron (mg)**	2.8 (16%)	1.7 (9%)	3.9 (22%)	9.3 (52%)	3.0 (17%)
**Calcium (mg)**	31 (2%)	27 (2%)	22 (2%)	17 (1%)	14 (1%)
**Magnesium (mg)**	114 (27%)	119 (28%)	130 (31%)	137 (33%)	114 (27%)
**Phosphorus (mg)**	290 (23%)	188 (15%)	280 (22%)	220 (18%)	285 (23%)
**Manganese (mg)**	1.2 (52%)	1.5 (65%)	1.7 (74%)	1.9 (83%)	1.6 (70%)
**Zinc (mg)**	2.4 (22%)	2.0 (18%)	2.6 (24%)	2.3 (21%)	1.7 (15%)
**Potassium (mg)**	250 (5%)	152 (3%)	281 (6%)	210 (4%)	195 (4%)
**Sodium (mg)**	6 (<1%)	4 (<1%)	6 (<1%)	4 (<1%)	5 (<1%)
**Thiamin – B1 (mg)**	0.59 (49%)	0.25 (21%)	0.33 (28%)	0.30 (25%)	0.35 (30%)
**Riboflavin – B2 (mg)**	0.11 (8%)	0.09 (7%)	0.10 (8%)	0.08 (6%)	0.10 (8%)
**Niacin – B3 (mg)**	3.2 (20%)	2.0 (13%)	3.5 (22%)	2.5 (16%)	4.0 (25%)
**Folate (μg)**	15 (4%)	18 (5%)	20 (5%)	30 (8%)	35 (9%)

## 8 Future prospects and concluding remark

Minor millets can be considered ‘superfoods’ or ‘smart foods’ because they are a powerhouse of nutrition and can also offer sustainability from ecological and socioeconomic standpoints. In comparison to major cereals, minor millets have the ability to fulfil the immediate requirement of global food security in various ways. The impact of external factors, such as climate and rainfall, is marginal on the productivity of minor millets, ensuring consistency in income for farmers. The cultivation of minor millets has shown a reduced dependency on various pesticides, synthetic fertilizers, and insecticides, whereas the cultivation of major cereals (wheat and rice) is reported to increase the global burden of carbon equivalent emissions (CEE). It has also been noted that the carbon footprints of major cereals are comparatively higher than minor millets. The majority of the global population relies on rice, wheat, or maize as their staple food, and overreliance on these crops has led to a crisis with limited access to food for the marginal population. The lack of diversity and unequal distribution of dependency among crops deleteriously impacted production and the supply chain during the COVID-19 pandemic, compromising the nutritional status of humankind. The use of minor millets can contribute to enhancing food diversity and also reduce the burden on major cereals due to over-dependency. However, ideation of crop improvement strategies is still in a nascent stage. The identification and utilisation of potential minor millet grains require attention, particularly in terms of adaptability to the environment, market value of production, taste, texture, and value-added product development. The climate-resilient, nutritive, and nutraceutical value of minor grains has been widely studied, indicating that they are capable of serving as staple crops in many hunger hotspots identified by the UN-FAO and UN-WFP. However, information on the production and yield of individual minor millet species is not specifically available. These data are required for the extensive cultivation of minor grains. Novel approaches are necessary to spread the significance of minor millets among the population in terms of cultivation and nutritional value, ensuring their successful implementation.

## CRediT authorship contribution statement

**Moumita Das:** Writing – review & editing, Writing – original draft, Methodology, Investigation, Formal analysis, Conceptualization. **Arpita Das:** Writing – review & editing, Writing – original draft, Methodology, Investigation, Conceptualization. **Harsh B. Jadhav:** Writing – review & editing, Writing – original draft, Methodology, Investigation, Conceptualization. **Dheeraj Kumar:** Writing – review & editing, Writing – original draft, Methodology, Investigation, Conceptualization. **Aayeena Altaf:** Writing – review & editing, Writing – original draft, Methodology, Investigation, Conceptualization. **Pankaj B. Pathare:** Writing – review & editing, Methodology, Investigation, Conceptualization. **Robert Mugabi:** Writing – review & editing, Supervision, Methodology, Conceptualization.

## Declaration of competing interest

The authors declare that they have no known competing financial interests or personal relationships that could have appeared to influence the work reported in this paper.

## Data Availability

No data was used for the research described in the article.
